# Facial Age Aftereffects Provide Some Evidence for Local Repulsion (But None for Re-Normalisation)

**DOI:** 10.1068/i0725jc

**Published:** 2015-04-01

**Authors:** Katherine R. Storrs

**Affiliations:** School of Psychology, The University of Queensland, St Lucia, Queensland, Australia

**Keywords:** adaptation, face perception, normalisation, opponent coding, visual aftereffects

## Abstract

Face aftereffects can help adjudicate between theories of how facial attributes are encoded. O'Neil and colleagues (2014) compared age estimates for faces before and after adapting to young, middle-aged or old faces. They concluded that age aftereffects are best described as a simple re-normalisation—e.g. after adapting to old faces, all faces look younger than they did initially. Here I argue that this conclusion is not substantiated by the reported data. The authors fit only a linear regression model, which captures the predictions of re-normalisation, but not alternative hypotheses such as local repulsion away from the adapted age. A second concern is that the authors analysed absolute age estimates after adaptation, as a function of baseline estimates, so goodness-of-fit measures primarily reflect the physical ages of test faces, rather than the impact of adaptation. When data are re-expressed as aftereffects and fit with a nonlinear “locally repulsive” model, this model performs equal to or better than a linear model in all adaptation conditions. Data in O'Neil et al. do not provide strong evidence for either re-normalisation or local repulsion in facial age aftereffects, but are more consistent with local repulsion (and exemplar-based encoding of facial age), contrary to the original report.

The properties of a face can seem to change depending on what other faces you have seen recently. For example, after looking at an old face, participants tend to rate middle-aged faces as being younger than they had initially (Schweinberger et al., 2010). Face aftereffects may result from neural adaptation of channels encoding facial attributes and, if so, studying the patterns of biases induced by adaptation might help reveal the number and selectivity of such channels (Webster & MacLeod, 2011).

There are two prominent theories concerning how facial attributes are encoded. According to exemplar-based proposals, multiple channels encode each attribute, with each channel selective for a particular value of that attribute, and no value playing a special role (Lewis, 2004; Valentine, 1991). According to norm-based proposals, channels are selective for the ways in which a face differs from a perceptual “norm” (e.g. the average face), which is constantly updated according to recent experience (Rhodes et al., 2005; Valentine, 1991). In simulations, these two encoding schemes predict markedly different patterns of adaptation-induced aftereffect (Storrs & Arnold, 2012; Webster & MacLeod, 2011). Exemplar-based encoding can predict a locally repulsive pattern of biases after adaptation, like that found after adapting to simple spatial properties such as orientation and spatial frequency (e.g. Blakemore, Nachmias & Sutton, 1970; Mitchell & Muir, 1976; Nemes, Whitaker, Heron, & McKeefry, 2011; Seriès, Stocker, & Simoncelli, 2009. See Figure 1, left). Norm-based encoding can instead predict re-normalisation, involving a uniform bias at all points along the test continuum (see Figure 1, right).

Despite the qualitative differences between local repulsion and re-normalisation, it is not clear which best describes face aftereffects. The two proposals can only be clearly dissociated by measuring changes in perception at the adapting face and for more “extreme” test faces, and this is difficult to do using common methods such as binary classification tasks (Storrs & Arnold, 2012). In a recent paper, O'Neil, Mac, Rhodes, & Webster. (2014) attempted to overcome this problem by measuring aftereffect patterns along a natural facial dimension for which people can easily report the appearance of *any* test face, using a reasonably precise and reliable numerical value: age.

In O'Neil et al. (2014), age estimates for 80 test faces were collected before adaptation, and again after adapting to a sequence of young, middle-aged or old faces. This provides a rich dataset, from which the perceptual change induced at any point along the age continuum, after each type of adaptation, can in principle be measured. The authors argued that the changes in age ratings after adaptation were best described as a uniform shift in perceived age across all test faces, and therefore that these data support norm-based theories of facial encoding.

**Figure 1.**
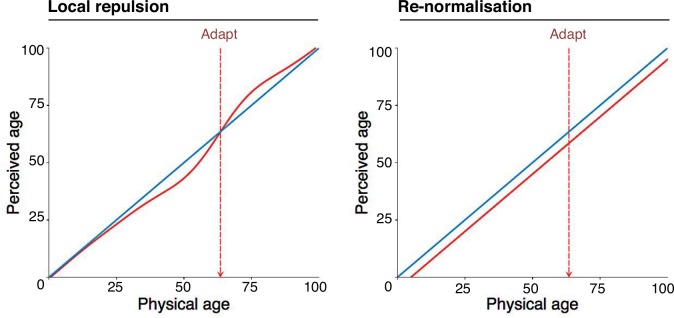
A schematic illustration of the predicted changes in perceived age after adapting to a moderately old face, if facial age aftereffects follow (left) a locally repulsive or (right) a re-normalising pattern. Before adaptation (blue curves), perceived age may match physical age. After adaptation (red curves), local repulsion predicts that the apparent age of the adapting face will not change, but younger faces will look exaggeratedly young, and older faces will look exaggeratedly old. Re-normalisation predicts that the adapting face and all other faces will appear younger by approximately the same amount.

However, there are two issues related to this conclusion. First, and most importantly, the authors presented and analysed fits to their data from a regression model containing only a linear term. This straight-line model well captures the predictions of a uniform re-normalisation (a shift in the intercept with no change in slope), but it doesn't well capture the predictions of a local repulsion. The authors proposed that local repulsion should manifest as a change in both the intercept and the slope of the best fitting line after adaptation, relative to baseline. This might reasonably describe shifts predicted by a very broadly tuned local repulsion, but since the tuning of any hypothetical local repulsion as a function of physical age is unknown, a linear model cannot comprehensively capture the local repulsion hypothesis. The authors note that including higher order terms in the linear regression model accounted for negligible additional variance. However, models with such terms (e.g. quadratic or cubic functions) also fail to capture the local-repulsion hypothesis. Below I compare the linear fits in the original report to an alternative model—a first derivative of a Gaussian function—which well describes the locally repulsive aftereffect pattern found following adaptation to simpler spatial patterns, such as tilt and spatial frequency (e.g. Blakemore et al., 1970; Mitchell & Muir, 1976; Nemes et al., 2011; Sèries et al., 2009).

Second, it is unclear in the original report how well model fits capture *aftereffect* patterns, as the authors performed regression on *absolute* age estimates for each test face after adaptation, as a function of absolute age estimates for each test face before adaptation. Unsurprisingly, post-adaptation age ratings were highly correlated with pre-adaptation ratings. O'Neil et al. specify that physical age accounts for ~94% of the variance in all regressions involving post-adaptation age ratings, so only minimal variance was left to be accounted for via the effects of adaptation. The linear model reported in O'Neil et al. explains ~98–99°% of the variance in post-adaptation age ratings, but it is important also to know how well the model explains the relatively minor *shifts* in age ratings induced by adaptation.

Since we are interested in the aftereffect pattern, it is more appropriate to analyse *differences* in estimated age for each test face before versus after adaptation. In Figure 1, I have re-expressed these data (available in the Supplementary data file of O'Neil et al. (2014)) in terms of the shift in average age estimate for each test face, as a function of the pre-adaptation average age estimate for that face. I fit each dataset independently with a linear model (blue lines) and with a nonlinear Gaussian-derivative model (red curves), in Matlab.

**Figure 2.**
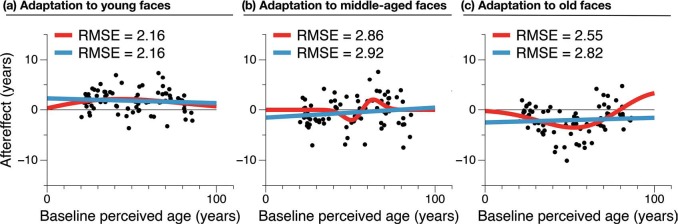
Average shift in estimated age for each test face, relative to its estimated age at baseline, after (a) adapting to young faces, (b) middle-aged faces or (c) old faces. Aftereffect data were fitted with a linear model (blue lines) and a first derivative of a Gaussian function (red curves). Root mean squared error (RMSE) is shown for each fit, and additional details are provided in the main text. All graphs and analyses are based on mean age ratings as given in the Supplementary data file of O'Neil et al. (2014).

Across all three datasets, neither model provided a good fit to aftereffect data (see Figure 2). For aftereffects induced by adapting to Young faces, linear regression *R*^2^ = 0.008, explaining less than 1% of the variance in aftereffect data. Root mean squared error (RMSE) of both the linear and nonlinear models^1^ = 2.16 years. For Middle-Aged adaptation, model fits are again poor and similar: linear model R^2^ = 0.019 (RMSE = 2.92 years) and nonlinear model RMSE = 2.86 years. For Old adaptation, linear model *R*^2^ = 0.005 (RMSE = 2.82 years) and nonlinear model RMSE = 2.55 years. Since the two models are not nested, it is not appropriate to compare them on the basis of an *F*-ratio. An alternative metric is the Bayesian Information Criterion (BIC) which provides unbiased estimates of goodness-of-fit for nonlinear models (Spiess & Neumayer, 2010) and contains a term to penalise more complex models. The lowest BIC value indicates the model that fits the data best with the fewest parameters, and the magnitude of the difference in BIC values between two models indicates the strength of evidence in favour of one over the other. For the Young adaptation data, linear model BIC = 355 and nonlinear model BIC = 357. According to the conventions suggested by Raftery (1995), a difference in BIC of 2 or less should not be considered evidence in favour of either model. For Middle-Aged adaptation, linear model BIC = 403 and nonlinear model = 402 (again a negligible difference). For Old adaptation, linear model BIC = 398, and nonlinear model BIC = 384. A BIC difference of >10 constitutes “very strong” evidence (Raftery, 1995), here in favour of the nonlinear local-repulsion model.

This new analysis makes two points. First and foremost, the shifts in age ratings for each test face are highly variable, and neither model provides a good fit to the data. The best linear fit explains less than 2% of the variance in aftereffect data for each test face. Second, if we were to attempt to compare the two models, two of the three datasets provide no evidence in favour of either model, and in the third, a Gaussian-derivative model (capturing a locally repulsive pattern of biases) provides a better fit to the aftereffect data than does a linear model (and is judged as superior using a metric which penalises this model for having one more parameter than the linear model).

The source of the variability in the age aftereffect across test faces is unknown. One possibility is that different test faces interacted differently with the series of adapting faces; for example because some test faces share many features with members of the adapting series while other test faces share few features with the adaptors. Aftereffects along one facial dimension are known to be largest when test and adapting stimuli are similar along other dimensions (Little, DeBruine, Jones, & Waitt, 2008). Such exemplar-specific effects may arise either from interactions between local properties of the adapting and test images (Dickinson, Almeida, Bell, & Badcock, 2010), or from the overlap between test and adapting faces in higher-level representations.

In conclusion, the pattern of perceptual changes induced by age adaptation is far from clear in these data. Where there is evidence for either hypothesis, they more strongly support the local-repulsion hypothesis (and exemplar-based encoding), than re-normalisation (and norm-based encoding), contrary to the title claim of O'Neil et al. (2014).

## Footnotes

1 R^2^ does not have a clear interpretation for nonlinear models (see Spiess & Neumayer, 2010).
